# PyCoM: a python library for large-scale analysis of residue–residue coevolution data

**DOI:** 10.1093/bioinformatics/btae166

**Published:** 2024-03-26

**Authors:** Philipp E Glass, Sabriyeh Alibai, Alessandro Pandini, Sarath Chandra Dantu

**Affiliations:** Department of Computer Science, Brunel University London, Uxbridge UB8 3PH, United Kingdom; Department of Computer Science, Brunel University London, Uxbridge UB8 3PH, United Kingdom; Department of Computer Science, Brunel University London, Uxbridge UB8 3PH, United Kingdom; Department of Computer Science, Brunel University London, Uxbridge UB8 3PH, United Kingdom

## Abstract

**Motivation:**

Computational methods to detect correlated amino acid positions in proteins have become a valuable tool to predict intra- and inter-residue protein contacts, protein structures, and effects of mutation on protein stability and function. While there are many tools and webservers to compute coevolution scoring matrices, there is no central repository of alignments and coevolution matrices for large-scale studies and pattern detection leveraging on biological and structural annotations already available in UniProt.

**Results:**

We present a Python library, PyCoM, which enables users to query and analyze coevolution matrices and sequence alignments of 457 622 proteins, selected from UniProtKB/Swiss-Prot database (length ≤ 500 residues), from a precompiled coevolution matrix database (PyCoMdb). PyCoM facilitates the development of statistical analyses of residue coevolution patterns using filters on biological and structural annotations from UniProtKB/Swiss-Prot, with simple access to PyCoMdb for both novice and advanced users, supporting Jupyter Notebooks, Python scripts, and a web API access. The resource is open source and will help in generating data-driven computational models and methods to study and understand protein structures, stability, function, and design.

**Availability and implementation:**

PyCoM code is freely available from https://github.com/scdantu/pycom and PyCoMdb and the Jupyter Notebook tutorials are freely available from https://pycom.brunel.ac.uk.

## 1 Introduction

Cellular function and development rely on intricate inter- and intrabiomolecular interactions. Genetic mutations can disrupt these interactions, compromising critical functions essential for life. A known mechanism of protection is the emergence of compensatory changes that preserve the network of interactions critical for the biological function recovering disrupted functions (Agozzino *et al.* 2020).

Since the 1960s, several studies have focused on inter-residue evolutionary relationships using various experimental and theoretical approaches, leading to the formulation of mathematical models of residue–residue coevolution (De Juan *et al.* 2013, Ochoa and Pazos 2014). Breakthroughs in the computational implementation of those models made it possible to develop powerful bioinformatics tools which can predict intra- and interstructural contacts in proteins (Morcos *et al.* 2011, Marks *et al.* 2012, Hopf *et al.* 2014), unveil protein function (Salinas and Ranganathan 2018), inform protein design (Russ *et al.* 2020), predict effects of pathogenic mutations on protein function and fold (Hopf *et al.* 2017, Kim *et al.* 2019), decode coevolution events in proteins (Ochoa and Pazos 2014), and most recently to enable large-scale AI-driven predictions of protein structures from sequence (Baek *et al.* 2021, Jumper *et al.* 2021).

The most widely used workflow to quantify pairwise coevolutionary relationships for a protein sequence requires, first, the generation of a multiple sequence alignment of homologous proteins followed by application of a method to estimate statistical coupling, like the statistical coupling analysis (SCA) (Halabi *et al.* 2009), or a variant of direct coupling analysis (Morcos *et al.* 2011, Ekeberg *et al.* 2014, Figliuzzi *et al.* 2018), EVcouplings (Hopf *et al.* 2019), GREMLIN (Kamisetty *et al.* 2013), or CCMpred (Seemayer *et al.* 2014). The final output is a pairwise coevolution scoring matrix. There are many command-line tools and web servers to calculate coevolution matrices, as well as two databases with precomputed results for ∼7000 human proteins (EVCouplings) and ∼9846 bacterial proteins (GREMLIN) (Supplementary Table S1). However, there is no integrated resource that can offer: (i) a database of precalculated coevolution matrices for most of UniProtKB/Swiss-Prot database (Boutet *et al.* 2016), (ii) an easy-to-use API to run complex queries on coevolution data based on structural and biological features recorded in UniProtKB/Swiss-Prot, and (iii) an effective way to develop user-designed workflows for large-scale analysis of coevolution data and sequence alignments.

Data accessibility is key to improving existing methods and developing new machine learning software tools for this domain (Xu and Jackson 2019, Wilson *et al.* 2021). To this end we have: (i) created a database of coevolution matrices (PyCoMdb), with access to corresponding sequence alignments, for ∼457 000 proteins from UniProt/Swiss-Prot, (ii) developed a Python library to mine, access and visualize the open-source database of coevolution matrices (PyCoM) and sequence alignments, and (iii) provided template workflows for large-scale analysis using Jupyter Notebooks.

## 2 Materials and methods

We developed PyCoM for the large-scale analysis of residue–residue coevolution matrices and their corresponding sequence alignments. We created an associated data resource covering the largest precompiled collection of residue-level protein coevolution data (PyCoMdb). The PyCoM library provides three ways of interaction with PyCoMdb: (i) on a locally downloaded copy, (ii) via remote API through a Flask wrapper for PyCoM, or (iii) using a web API interface (https://pycom.brunel.ac.uk/api/spec/). Quick guides for the three modes are available from https://pycom.brunel.ac.uk/gettingstarted.html.

PyCoMdb contains residue–residue coevolution scoring matrices for unique protein sequences from UniProtKB/Swiss-Prot version 2022_03. Due to computational costs, only sequences up to 500 residues in length were included. The database has 80% coverage of the UniProtKB/Swiss-Prot database, i.e. 457 622 proteins with 377 409 unique sequences. PyCoMdb has information on 24 UniProt annotated features that can be queried using 29 keywords (Supplementary Table S2). Data are split across two subdatabases: (i) annotation of biological and functional features (Supplementary Tables S2 and S3) of each protein is stored in a SQLite database (*pycom.db*) and (ii) a compressed HDF5 database (*pycom.mat*) storing precomputed coevolution matrices of proteins from *pycom.db*. Coevolution matrices were computed for each unique protein sequence based on the protocol from Kamisetty *et al.* 2013 using HH-suite3 package (Steinegger *et al.* 2019) for homology search and sequence alignment and CCMpred (Seemayer *et al.* 2014) for calculation of pairwise coevolution scores (full details are available in the Methods section of Supplementary Data). CCMpred was chosen for its speed (35–113× faster than other tools) and accuracy in contact prediction (Seemayer *et al.* 2014). Each sequence alignment file can be downloaded individually, and this is demonstrated in the tutorials.

PyCoM implements the following user-facing functionalities: (i) PyCom—queries a local or remote PyCoM database, returns the search results as a pandas dataframe, loads additional biological features (Supplementary Table S3), and coevolution matrices; (ii) CoMAnalysis—contains helper functions to process, analyze, normalize coevolution matrices and export top-scoring coevolution residue pairs; (iii) *pdb2res* & *pdb_analysis—*a lightweight PDB parser and analysis tool, to aid comparison to experimental structures or AlphaFold (Jumper *et al.* 2021) predicted protein structures.

Description of the data, documentation, tutorials on how to use PyCoM, the database files, and access to the web API interface are available from the PyCoM website (https://pycom.brunel.ac.uk) and the GitHub repository (https://github.com/scdantu/pycom).

## 3 Results

**Figure 1.**
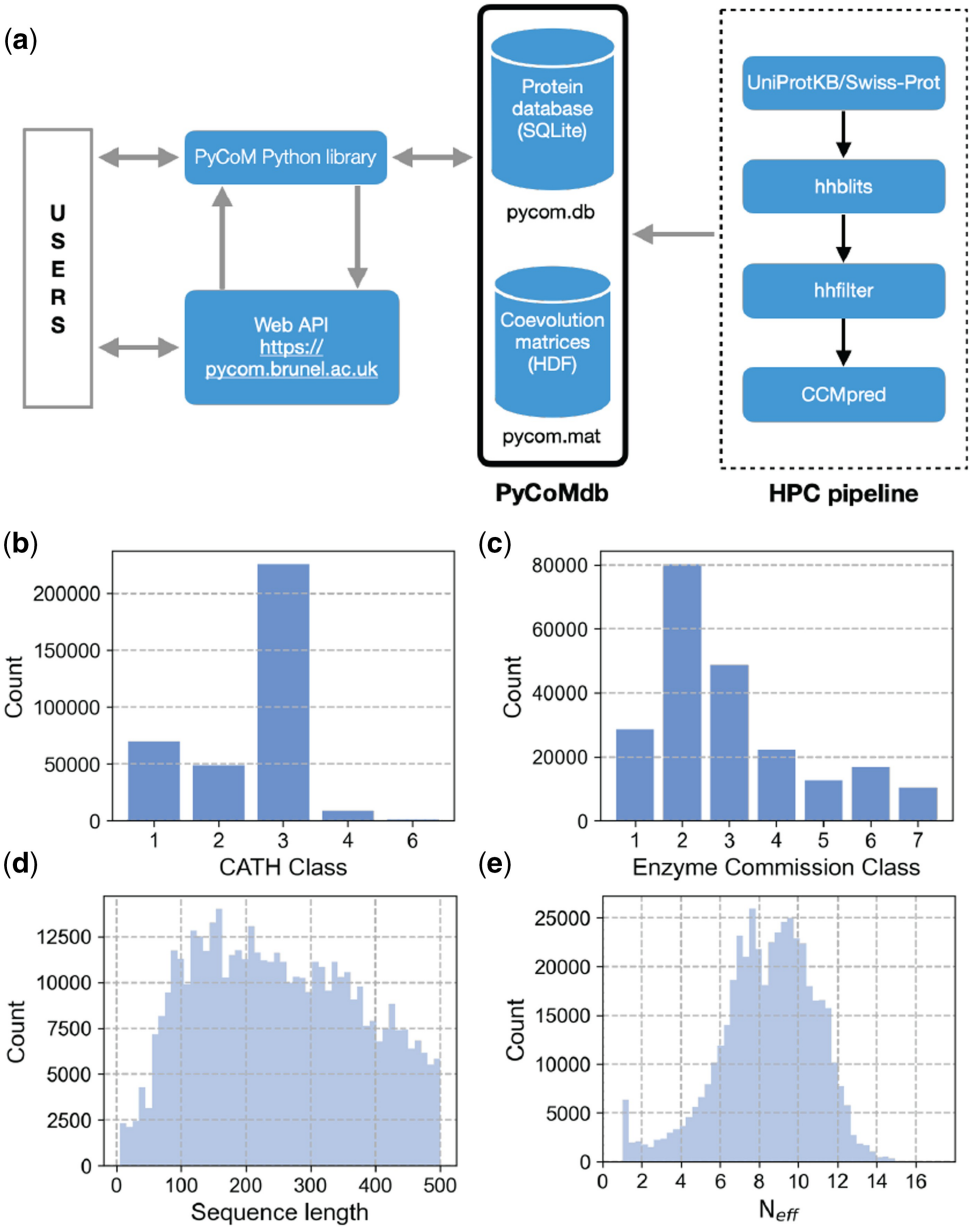
PyCoMdb contains annotated information of 457 622 proteins with corresponding coevolution matrices. (a) Schema showing how the users, via PyCoM library interact directly with either the local PyCoM database (PyCoMdb) or the remote PyCoMdb hosted on pycom.brunel.ac.uk server. Further, the user can directly query the database using the web API. The number of proteins by CATH class and Enzyme Commission class are shown in (b) and (c) panels; (d) and (e) are the distributions of protein sequence length and the effective sequence depth (*N*_eff_) of all the alignments in PyCoMdb. Users will find the Python code to generate panels (b–e) in Supplementary Material 04_Paper_Images.

PyCoMdb includes information on 457 622 proteins, with a mean protein length of 251 and representatives from all CATH and Enzyme commission classes (Fig. 1). 91% of the proteins (416 587) have more than five effective sequences ($N_{\mathrm{eff}}$) in the alignment, which is the suggested threshold for statistically significant estimates of residue coevolution scores (Fig. 1e).

A typical workflow with PyCoM is shown in Supplementary Fig. S1. After the user constructs a query dictionary, the pycom object executes the query and returns a pandas dataframe with information on 12 protein features (Supplementary Table S2) allowing the user to analyze the statistics of the search results and if required, refine them further using additional properties, loading more structural or biological information available (Supplementary Table S3) using the data loader object. Following refinement of the query, users can load coevolution matrices into a dataframe, analyze the matrices (scaling and normalization are supported), and export the top-scoring residue-pairs list. As the search results and the matrix data are in a pandas dataframe, users can further benefit from pandas’ analysis and visualization functions. Jupyter Notebooks demonstrating example use of PyCoM with multiple use cases, including interpretation of coevolution scores and analyzing sequence alignments are available from the PyCoM website, the GitHub repository, and are included as Supplementary Material.

## 4 Conclusion

We have presented a Python library that allows for the searching of proteins and associated sequence alignments and coevolution matrix data using controlled vocabulary providing coverage for 9 of the 10 categories from UniProtKB/Swiss-Prot. The availability of UniProt-level data and analysis tools can support the bioinformatics community in effectively using residue coevolution data to unveil the biological role of individual residues in protein function and stability, to develop novel methods in computational protein design and to validate new coevolution-based methods. Novice users will benefit from the easy-to-use friendly framework of Jupyter Notebooks for the analysis of coevolution matrices and through Web API we have facilitated data access for expert users. This unique resource will aid in the development of data-driven machine learning models for coevolution, epistasis, protein structure and function predictions, and protein design. (Luo *et al.* 2021, Shin *et al.* 2021, Cagiada *et al.* 2023). In the next iteration, we will expand the database further to include proteins >500 residues and include coevolution matrices from multiple methods.

## Supplementary Material

btae166_Supplementary_Data

## Data Availability

The data underpinning this article can be accessed from Brunel University London’s data repository under CC BY license: Coevolution matrix database https://brunel.figshare.com/articles/dataset/PyCoM_ProCoM_Database_of_coevolution_matrices/23735613 and protein database https://brunel.figshare.com/articles/dataset/PyCoM_ProCoM_Curated_UniProt_protein_database/23733309.
