# Supplementary material for: PyCoM: a python library for large-scale analysis of residue–residue coevolution data
Source: Bioinformatics. 2024 Mar 26;40(4):btae166. doi: 10.1093/bioinformatics/btae166 (PMC11009027; doi:10.1093/bioinformatics/btae166)
Supplement: btae166_Supplementary_Data [file btae166_supplementary_data.zip › 02_UseCases.pdf]

Use cases

Here are some more examples on how you can query the PyCoMdb using either a keyword or combination of keywords, followed by one example of how to interpret coevolution matrix data.

Query by:

1. UniProtID
2. Sequence
3. EC/CATH
4. Disease
5. Multiple Keywords
6. Interpreting coevolution matrix

Import all the essential PyCoM classes...

In [ ]:
```
# importing all usefull classes from PyCoM
from pycom import PyCom, ProteinParams,CoMAnalysis
# other useful libraries
import pandas as pd
import numpy as np
import igraph as ig
import matplotlib.pyplot as plt
```

Setup the paths to the local database files if you are using PyCoM (local) i.e. with PyCoMdb on your computer.

In [ ]:
```
#set the path to the database
database_folder_path="/Volumes/mason/Work/Sarath/Research/pycom/"
#matrix file name and path
file_matrix_db = database_folder_path+"pycom.mat"
#protein database file name and path
file_protein_db= database_folder_path+"pycom.db"
```

In [ ]:
```
obj_pycom = PyCom(db_path=file_protein_db, mat_path=file_matrix_db)
```

.. or you can use PyCoM with the remote version of PyCoMdb, uncomment or comment the code as per the right scenario.

In [ ]:
```
#obj_pycom=PyCom(remote=True)
```

UniProtID

Using the UniProtID

In [ ]:
```
query_parameters={ProteinParams.ID:"Q65209"}
# use for remote mode
#entries_data_frame=obj_pycom.find(query_parameters,page=1)
entries_data_frame=obj_pycom.find(query_parameters)
entries_data_frame
```

Out[ ]:

	uniprot_id	neff	sequence_length	
**0**	Q65209	6.062	141	MGNKESKYLEMCSEEAWLNIPNIFKCIFIRKLFYNKV

Sequence

Using the **complete** protein sequence, *using partial protein sequence will not yield any or incorrect result.*

In [ ]:
```
query_parameters={ProteinParams.SEQUENCE:"MGNKESKYLEMCSEEAWLNIPNIFKCIFIRK
entries_data_frame=obj_pycom.find(query_parameters)
entries_data_frame
```

Out[ ]:

uniprot_id	neff	sequence_length	sequence	organism_id	helix_frac	turn_frac

EC and CATH

Using a combination of Enzyme Commission number and CATH ID.

In [ ]:
```
#
query_parameters={ProteinParams.CATH:"1.*",
                  ProteinParams.ENZYME:"3.2.*"
                 }
entries_data_frame=obj_pycom.find(query_parameters)
entries_data_frame
```

Out[ ]:

	uniprot_id	neff	sequence_length	
**0**	A0A0S2UQQ5	8.393	380	MNITGKGAYDTGTYANLFQRSGYREDEIKA
**1**	A0A168WVR6	8.987	382	MDESNLLQGISMIDLRSPDAILSDYAKRY
**2**	A0R567	8.089	293	MSISPVELLSWYDHARRDLPWRRPGVSAW
**3**	A1A048	8.357	379	MTNATDTNKTLGESMFAQCGYAQDAIDKRV
**4**	A1D1W1	7.956	493	MHLPSLSVALALVSSSLALPQTVLPESDVS
**...**	...	...	...	
**149**	Q9YDP0	10.113	223	MLFLDKGRIEALRRRLIEWYRVYGDKDLP
**150**	Q9YE60	9.077	288	MAQRVRWERVERVAEAFSRLSIGEVLGFE
**151**	Q9ZXB7	9.295	177	MNAKIRYGLSAAVLALIGAGASAPEILDQ
**152**	T2KLZ3	11.203	400	MRKLVYLVLVLGLTFLNVRCKSETKQNKK
**153**	T2KPL9	11.978	377	MKNQALKILTLCVLVGSAMSLKLYAQKGL

154 rows × 12 columns

We have found 153 proteins that match our search criteria i.e., proteins from CATH class `1.*` and EC class `3.2.*`.

Disease

Getting proteins linked to a particular disease.

In [ ]:

```
query_parameters={ProteinParams.DISEASE:"Cancer"}
entries_data_frame=obj_pycom.find(query_parameters)
entries_data_frame
```

Out[ ]:

	uniprot_id	neff	sequence_length	
**0**	O00358	5.065	373	MTAESGPPPPQPEVLATVKEERGETAAGAGVP
**1**	O15105	6.671	426	MFRTKRSALVRRLWRSRAPGGEDEEEGAGGGG
**2**	O43502	9.997	376	MRGKTFRFEMQRDLVSFPLSPAVRVKLVSA
**3**	O43542	10.237	346	MDLDLLDLNPRIIAAIKKAKLKSVKEVLHFS
**4**	O75771	10.597	328	MGVLRVGLCPGLTEEMIQLLRSHRIKTVVDL
**5**	P01111	12.817	189	MTEYKLVVVGAGGVGKSALTIQLIQNHFVD
**6**	P01112	12.841	189	MTEYKLVVVGAGGVGKSALTIQLIQNHFVD
**7**	P01116	12.626	189	MTEYKLVVVGAGGVGKSALTIQLIQNHFVD

**8**	P04637	5.749	393	MEEPQSDPSVEPPLSQETFSDLWKLLPENNVL
**9**	P08118	7.621	114	MNVLLGSVVIFATFVTLCNASCYFIPNEGVPG
**10**	P10914	5.137	325	MPITRMRMRPWLEMQINSNQIPGLIWINKEE
**11**	P16422	6.971	314	MAPPQVLAFGLLLAAATATFAAAQEECVCENY
**12**	P21757	10.961	451	MEQWDHFHNQQEDTDSCSESVKFDARSMTAL
**13**	P31749	10.926	480	MSDVAIVKEGWLHKRGEYIKTWRPRYFLLKN
**14**	P43699	6.301	371	MSMSPKHTTPFSVSDILSPLEESYKKVGMEGG
**15**	P50539	6.898	228	MERVKMINVQRLLEAAEFLERRERECEHGYA
**16**	P60484	9.037	403	MTAIIKEIVSRNKRRYQEDGFDLDLTYIYP
**17**	P62070	12.754	204	MAAAGWRDGSGQEKYRLVVVGGGGVGKSAL
**18**	P84022	7.019	425	MSSILPFTPPIVKRLLGWKKGEQNGQEEKWC
**19**	Q06609	9.769	339	MAMQMQLEANADTSVEEESFGPQPISRLEQC
**20**	Q14982	13.369	345	MGVCGYLFLPWKCLVVVSLRLLFLVPTGVPVR
**21**	Q15198	11.902	375	MKVWLLLGLLLVHEALEDVTGQHLPKNKRPK
**22**	Q5HYI8	11.991	236	MASLDRVKVLVLGDSGVGKSSLVHLLCQNQVL
**23**	Q6UWZ7	6.040	409	MEGESTSAVLSGFVLGALAFQHLNTDSDTE
**24**	Q92826	7.266	284	MEPGNYATLDGAKDIEGLLGAGGGRNLVAHS
**25**	Q96BI1	11.891	424	MQGARAPRDQGRSPGRMSALGRSSVILLTY
**26**	Q99612	10.929	283	MDVLPMCSIFQELQIVHETGYFSALPSLEEY
**27**	Q9NZC7	11.052	414	MAALRYAGLDDTDSEDELPPGWEERTTKDGWV
**28**	Q9UBG3	5.694	495	MPQLLQNINGIIEAFRRYARTEGNCTALTR
**29**	Q9UNW1	9.554	487	MLRAPGCLLRTSVAPAAALAAALLSSLARC

That's 29 proteins with known association to cancer.

Multiple Keywords

In [ ]:
```
query_parameters={ProteinParams.CATH:"1.*",
                  ProteinParams.DISEASE:"Cancer"
                 }
entries_data_frame=obj_pycom.find(query_parameters)
entries_data_frame
```

Out[ ]:

	uniprot_id	neff	sequence_length	
0	O00358	5.065	373	MTAESGPPPPQPEVLATVKEERGETAAGAGVPGEA
1	P10914	5.137	325	MPITRMRMRPWLEMQINSNQIPGLIWINKEEMIFQ
2	Q96BI1	11.891	424	MQGARAPRDQGRSPGRMSALGRSSVILLTYVLA/

We found 3 proteins from CATH class `1.*` i.e., mainly alpha helical proteins. For help on CATH Heirarchy.

Interpreting coevolution matrix

Now let's get the coevolution matrices for these 3 proteins and understand how to interpret the data using one of the proteins `O00358`.

In [ ]:
```
# load the coevolution matrices
entries_data_frame=obj_pycom.load_matrices(entries_data_frame)
```

Coevolution matrix (C_i) for each protein has coevolution scores c_{ij} for all residue pairs `i` & `j`.

Residue pairs with coevolution score $c_{ij} \geq \langle C_i \rangle$ are considered significant, therefore C_i is scaled by average $\langle C_i \rangle$ as shown below:

$$S_i = \frac{C_i}{\langle C_i \rangle}$$

Note: All $c_{ij} \leq \langle C_i \rangle$ are set to `0`

To compare coevolution scores between two or more proteins, S_i are normalised (N_i) as shown below:

$$N_i = \frac{S_i}{max\langle S_i ... S_N \rangle}$$

In [ ]:
```
obj_com_analysis=CoMAnalysis()
# scale and normalise the coevolution matrices and add them to the datafr
entries_data_frame=obj_com_analysis.scale_and_normalise_coevolution_matri
```

There are three proteins in our data frame, listed below:

In [ ]:
```
#
entries_data_frame['uniprot_id']
```

Out[ ]:
```
0    O00358
1    P10914
2    Q96BI1
Name: uniprot_id, dtype: object
```

For the first protein, lets get the top scoring residues pairs from the 90th percentile:

In [ ]:
```
#for matrix in entries_data_frame['matrix_S']:
df_top_scoring_residues=obj_com_analysis.get_top_scoring_residues(entries
df_top_scoring_residues
```

Out[ ]:

	ResA	ResB	coevolution_score
**0**	81	134	3.114167
**1**	333	360	3.075198
**2**	1	8	2.830141
**3**	320	367	2.802745
**4**	232	345	2.790903
**...**	...	...	...
**6069**	13	27	1.406554
**6070**	196	281	1.406419
**6071**	70	172	1.406395
**6072**	194	249	1.406223
**6073**	185	224	1.406221

6074 rows × 3 columns

Residues 81 and 134 have the strongest coevolution signal/score of 3.11, i.e., there is a very high chance that a mutation in one has a resulted in a change in the other residue. For example studies please refer to:

1. Bai et al.
2. Hopf et al.
3. Akere et al.

It is also very interesting to check which residue is the most coevolving, i.e., involved in most number of strongly coevolving pairs and which residue is the least coevolving, i.e., is involved in the least number of pairs.

In [ ]:
```
df_residue_frequencies=obj_com_analysis.get_residue_frequencies(df_top_sc
df_residue_frequencies
```

Out[ ]:

	residueID	count
**0**	81	39
**1**	333	46
**2**	1	17
**3**	320	35
**4**	232	34
**...**	...	...
**364**	365	29
**365**	355	8
**366**	353	12
**367**	354	8
**368**	131	14

369 rows × 2 columns

In [ ]:
```
# saving the residue frequencies to a csv file
fname="output/csv/"+entries_data_frame['uniprot_id'][0]+"_res_freqs.csv"
df_residue_frequencies.to_csv(fname,index=False)
```

In [ ]:
```
#saving the top scoing residue pairs to a csv file
obj_com_analysis.save_top_scoring_residue_pairs(entries_data_frame,matrix
```

Let us compare if the range of the coevolution scores for the top 10% of the residue pairs is different or similar for the three proteins.

In [ ]:
```
list_top_residue_stats=[]
for uniprotid,matrix in zip(entries_data_frame['uniprot_id'],entries_data
    df_residue_pairs_scores=obj_com_analysis.get_top_scoring_residues(mat
    list_top_residue_stats.append(df_residue_pairs_scores['coevolution_sc
```

In [ ]:
```
my_color="#6495ED"
ticks_font=12
labels_font=14
```

In [ ]:
```
plt.figure(figsize=(4,3))
plt.boxplot(list_top_residue_stats)
plt.xticks(range(1, len(list_top_residue_stats) + 1), entries_data_frame[
plt.xlabel("UniProt ID",fontsize=labels_font)
plt.ylabel("Scaled Coevolution Score ",fontsize=labels_font-2)
plt.xticks(fontsize=ticks_font)
plt.yticks(fontsize=ticks_font)
plt.grid(axis='y',ls="--",lw=1)
```

```
plt.tight_layout()
plt.savefig("output/png/04_box_plot.png",dpi=300,transparent=True)
```

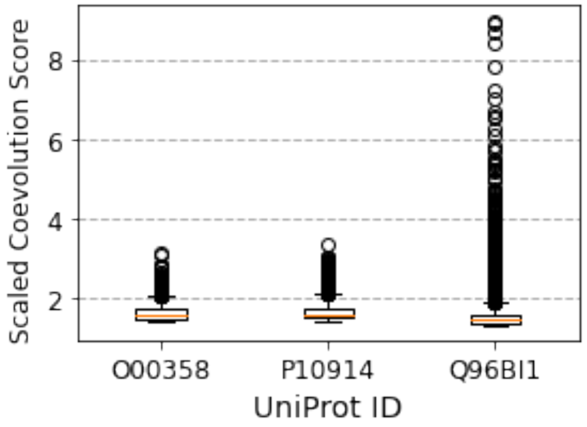

In [ ]:
```
plt.figure(figsize=(4,3))
plt.bar(range(0,len(list_top_residue_stats),1),
        height=[len(pair_list) for pair_list in list_top_residue_stats],
        color=my_color,
        tick_label=entries_data_frame['uniprot_id']
        )
plt.xlabel("UniProt ID",fontsize=labels_font)
plt.ylabel("Residue pairs (#)",fontsize=labels_font)
plt.xticks(fontsize=ticks_font)
plt.yticks(fontsize=ticks_font)
plt.grid(axis='y',ls="--",lw=1)
plt.tight_layout()
plt.savefig("output/png/04_bar_plot.png",dpi=300,transparent=True)
```

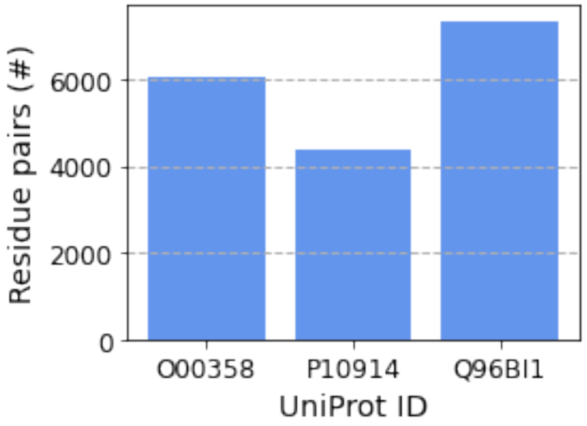

Based on the box plot the range of scaled coevolution scores is different between the three proteins and the bar plot, the number of residue pairs is also different.
