# Supplementary material for: PyCoM: a python library for large-scale analysis of residue–residue coevolution data
Source: Bioinformatics. 2024 Mar 26;40(4):btae166. doi: 10.1093/bioinformatics/btae166 (PMC11009027; doi:10.1093/bioinformatics/btae166)
Supplement: btae166_Supplementary_Data [file btae166_supplementary_data.zip › 03_Alignment_analysis.pdf]

Analysing alignment(s)

In [36]:
```
import requests
import re
import logomaker as lmaker
import numpy as np
```

Functions to download, read and transform alignment files

In [26]:
```
def download_alignment_files(uniprot_id):
    url="https://pycom.brunel.ac.uk/alignments/"
    file_name=uniprot_id+".aln"

    web_url=url+file_name

    try:
        response = requests.get(web_url, stream=True)

        # Check if the request was successful (HTTP status code 200)
        response.raise_for_status()
        if response.status_code == 200:
        # Open the file in binary write mode and save the content
            with open(file_name, 'wb') as file:
                for chunk in response.iter_content(chunk_size=8192):
                    file.write(chunk)

            print(f"File downloaded successfully and saved as '{file_name
    except requests.exceptions.RequestException as e:
        print(f"Error downloading the file: {e}")

def convert_to_fasta(aln_file_in,fasta_file_out):
    file_data = open(aln_file_in,'r')
    count=0
    out=""
    for line in file_data:
        out+=">SEQ_%d\n"%(count)
        out+="%s"%(line)
        count+=1
    f=open(fasta_file_out,'w')
    f.write(out)
    f.close()

def read_alignment_file(file_path):
    with open(file_path, "r") as file:
        content = file.read()
        sequences = re.findall(r'[A-Z\-]+', content)
    return sequences
```

Download the alignment file

In [27]:
```
#uniprot_id="P62070"
uniprot_id="P0C5H4"
# download the alignment file from PyCoM (pycom.brunel.ac.uk) server
download_alignment_files(uniprot_id)
```

```
File downloaded successfully and saved as 'P0C5H4.aln'.
```

Read the alignment file

In [28]:
```
# get the sequences from the alignment file
alignment_file_path = uniprot_id+".aln"
sequences = read_alignment_file(alignment_file_path)
```

Convert the sequence to FASTA format

In [29]:
```
# Generate sequence alignment compatible with https://alignmentviewer.org
# you can upload the fasta file to https://alignmentviewer.org/ for analy
convert_to_fasta("P0C5H4.aln","P0C5H4.fasta")
```

In [30]:
```
# number of sequences in the alignment file
len(sequences)
```

Out[30]:
```
23
```

Transfrom sequence list to frequency matrix

In [31]:
```
# convert the sequence list to a pandas dataframe with count's of each am
df_sequence_count_matrix=lmaker.alignment_to_matrix(sequences)
df_sequence_count_matrix
```

Out[31]:

pos	A	C	D	E	F	G	H	I	K	L	M	N	P	Q	R	S	T	V	W	Y
**0**	0	0	0	0	0	0	0	2	0	19	0	0	0	0	2	0	0	0	0	0
**1**	0	0	0	4	0	0	0	1	17	0	0	0	0	1	0	0	0	0	0	0
**2**	0	23	0	0	0	0	0	0	0	0	0	0	0	0	0	0	0	0	0	0
**3**	0	0	1	0	0	0	2	0	2	0	0	17	0	0	0	0	0	0	0	1
**4**	0	0	0	0	0	0	0	0	12	0	0	0	0	3	2	1	5	0	0	0
**5**	0	0	0	0	0	0	0	1	0	16	0	0	4	1	0	0	0	1	0	0
**6**	0	0	0	0	1	1	0	7	0	5	0	0	0	0	0	0	0	9	0	0
**7**	0	0	1	0	0	0	0	0	1	0	0	0	20	0	1	0	0	0	0	0
**8**	0	0	0	0	6	0	0	5	0	6	0	0	5	0	0	0	0	0	1	0
**9**	6	0	0	0	8	0	1	3	0	3	0	0	0	0	0	0	0	2	0	0
**10**	0	0	0	0	0	0	3	0	1	0	0	0	0	0	0	5	1	0	5	8
**11**	0	1	0	0	0	0	0	1	20	0	1	0	0	0	0	0	0	0	0	0
**12**	0	0	0	0	0	0	0	0	1	0	0	0	1	0	0	0	21	0	0	0
**13**	0	22	0	0	0	0	0	0	0	0	0	1	0	0	0	0	0	0	0	0
**14**	0	0	0	0	0	0	0	0	1	0	0	0	22	0	0	0	0	0	0	0
**15**	5	0	0	15	0	0	0	0	1	0	0	0	1	0	0	0	0	1	0	0
**16**	0	0	0	0	0	22	0	0	0	0	0	0	0	0	0	0	0	1	0	0
**17**	0	0	0	0	0	0	0	0	17	0	0	2	1	3	0	0	0	0	0	0
**18**	0	0	1	0	1	0	0	0	0	0	0	21	0	0	0	0	0	0	0	0
**19**	0	0	0	0	0	0	0	1	0	22	0	0	0	0	0	0	0	0	0	0

Plot the Logo images

In [32]:

```
# Based on tutorial from logo https://logomaker.readthedocs.io/en/latest/

seq_logo=lmaker.Logo(df_sequence_count_matrix,
            color_scheme="chemistry",
            font_name='Arial Rounded MT Bold')
seq_logo.style_xticks(anchor=0, spacing=2, rotation=45)
#seq_logo.highlight_position(p=4, color='gold', alpha=.5)
#seq_logo.highlight_position(p=26, color='gold', alpha=.5)

# style using Axes methods
seq_logo.ax.set_ylabel('Count')
seq_logo.ax.set_xlim([-1, len(df_sequence_count_matrix)])
```

Out[32]: (-1.0, 20.0)

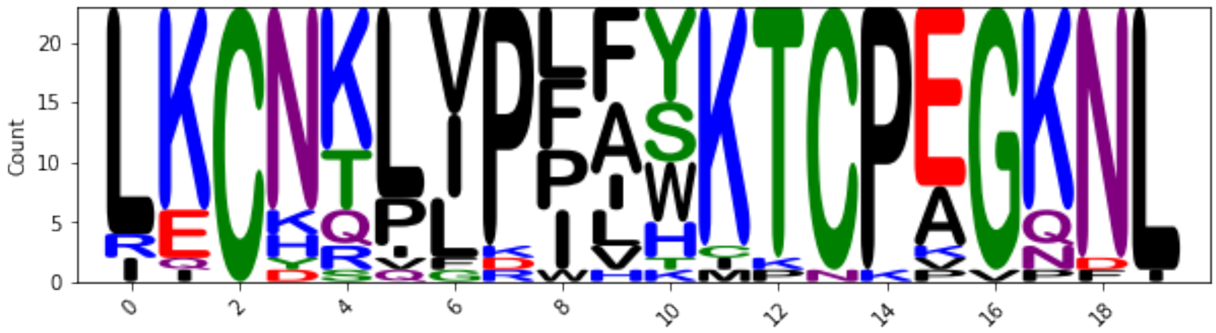

Matrix transformation examples

In [33]:
```
# convert frequency matrix to probability matrix
df_probability_matrix=lmaker.transform_matrix(df_sequence_count_matrix,no
# convert probability matrix to information matrix
df_information_matrix=lmaker.transform_matrix(df_probability_matrix,from_
```

Some more Logo images

In [34]:
```
df_information_matrix
```

Out[34]:

pos	A	C	D	E	F	G	H	I
0	0.000000	0.000000	0.000000	0.000000	0.000000	0.000000	0.000000	0.302734
1	0.000000	0.000000	0.000000	0.550845	0.000000	0.000000	0.000000	0.137711
2	0.000000	4.321928	0.000000	0.000000	0.000000	0.000000	0.000000	0.000000
3	0.000000	0.000000	0.130150	0.000000	0.000000	0.000000	0.260300	0.000000
4	0.000000	0.000000	0.000000	0.000000	0.000000	0.000000	0.000000	0.000000
5	0.000000	0.000000	0.000000	0.000000	0.000000	0.000000	0.000000	0.127339
6	0.000000	0.000000	0.000000	0.000000	0.104259	0.104259	0.000000	0.729811
7	0.000000	0.000000	0.154633	0.000000	0.000000	0.000000	0.000000	0.000000
8	0.000000	0.000000	0.000000	0.000000	0.562585	0.000000	0.000000	0.468821
9	0.526072	0.000000	0.000000	0.000000	0.701429	0.000000	0.087679	0.263036
10	0.000000	0.000000	0.000000	0.000000	0.000000	0.000000	0.268449	0.000000
11	0.000000	0.154633	0.000000	0.000000	0.000000	0.000000	0.000000	0.154633
12	0.000000	0.000000	0.000000	0.000000	0.000000	0.000000	0.000000	0.000000
13	0.000000	3.887218	0.000000	0.000000	0.000000	0.000000	0.000000	0.000000
14	0.000000	0.000000	0.000000	0.000000	0.000000	0.000000	0.000000	0.000000
15	0.619805	0.000000	0.000000	1.859416	0.000000	0.000000	0.000000	0.000000
16	0.000000	0.000000	0.000000	0.000000	0.000000	3.887218	0.000000	0.000000
17	0.000000	0.000000	0.000000	0.000000	0.000000	0.000000	0.000000	0.000000
18	0.000000	0.000000	0.165598	0.000000	0.165598	0.000000	0.000000	0.000000
19	0.000000	0.000000	0.000000	0.000000	0.000000	0.000000	0.000000	0.176692

In [35]:

```
seq_logo=lmaker.Logo(df_information_matrix,vpad=0.1,width=0.8,figsize=(21
seq_logo.style_xticks(anchor=0, spacing=2, rotation=45)
#seq_logo.highlight_position(p=4, color='gold', alpha=.5)
#seq_logo.highlight_position(p=26, color='gold', alpha=.5)

# style using Axes methods
seq_logo.ax.set_ylabel('Information (bits)')
seq_logo.ax.set_xticks(np.arange(0,len(df_sequence_count_matrix)+4,2))
seq_logo.ax.set_xlim([-1, len(df_sequence_count_matrix)])
```

Out[35]:

```
(-1.0, 20.0)
```

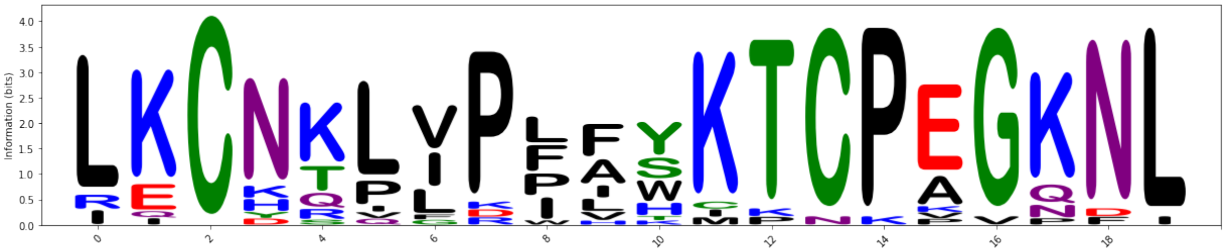
Information (bits)
4.0
3.5
3.0
2.5
2.0
1.5
1.0
0.5
0.0
0
2
4
6
8
10
12
14
16
18
