# Supplementary material for: PyCoM: a python library for large-scale analysis of residue–residue coevolution data
Source: Bioinformatics. 2024 Mar 26;40(4):btae166. doi: 10.1093/bioinformatics/btae166 (PMC11009027; doi:10.1093/bioinformatics/btae166)
Supplement: btae166_Supplementary_Data [file btae166_supplementary_data.zip › Supplementary_Information_v1.7_200324.docx]

**PyCoM: a python library for large-scale analysis of residue-residue coevolution data**

Philipp Bibik^1^, Sabriyeh Alibai^1^, Alessandro Pandini^1^, Sarath Chandra Dantu^1,*^

^1^Department of Computer Science, Brunel University London, Uxbridge, UB8 3PH, UK

*Corresponding author: [Sarath.dantu@brunel.ac.uk](mailto:Sarath.dantu@brunel.ac.uk)

**Supplementary Methods**

**Sequence alignment**

Multiple sequence alignment for the target sequence was generated using HH-suite package (Steinegger *et al.*, 2019) in two stages.

Stage 1

Using hhblits with the flags “-B 100000 -v 2 -n 4 -nodiff -maxfilt 100000 -maxseq 2000000” search for homologus sequences were performed using UniRef30_2022_02 database.

Stage 2:

Sequences were filtered using hhfilter to retaine sequences with 90% sequence identity (-id 90) and 75% coverage (-cov 75).

The alignment was filtered to retain only unique sequences using bash sort command to generate the input alignment for CCMpred (Seemayer *et al.*, 2014).

**Coevolution matrix calculation**

Coevolution matrix was calculated from the final alignment using CCMpred with number of iterations (-n) set to 75.

This entire pipeline was executed on JADE2 HPC system.

**Supplementary Table 1: List of software tools and webservers to perform protein coevolution analysis and databases with precomputed results.**

| **Command line tools** | | |
| --- | --- | --- |
| Name | Description | Website/github link |
| Direct Coupling Analysis (DCA) (Zerihun et al., 2020) | Identifies direct statistical couplings between pairs of positions in a protein sequence alignment | <https://github.com/KIT-MBS/pydca> |
| EVCouplings (Hopf et al., 2019) | Implements various methods for evolutionary couplings analysis, including DCA. | <https://github.com/debbiemarkslab/EVcouplings> |
| PSICOV(Jones et al., 2012) | Predicts residue-residue contacts from multiple sequence alignments using statistical coupling analysis | <https://github.com/psipred/psicov> |
| GREMLIN (Kamisetty et al., 2013) | Uses graphical models to infer evolutionary couplings from multiple sequence alignments | <https://github.com/sokrypton/GREMLIN> |
| Coevol (Lartillot and Poujol, 2011) | Analyzes protein co-evolution and predicts residue-residue contacts | <https://megasun.bch.umontreal.ca/People/lartillot/www/index.htm> |
| Mutual information (Martin et al., 2005) | Implements a mutual information-based method for the detection of residue-residue contacts. | <https://www.biotite-python.org/examples/gallery/sequence/residue_coevolution.html> |
| plmc (Hopf et al., 2017) | Probabilistic Latent Variable Models for Co-evolution | <https://github.com/debbiemarkslab/plmc> |
| MetaPSICOV 2.0(Jones et al., 2015) | Improved metapsicov for protein contact prediction | <https://github.com/psipred/metapsicov> |
| CCMpred (Seemayer et al., 2014) | CCMpred (Correlated Mutation Analysis for Multiple Protein Sequences) is a tool based on the direct coupling analysis (DCA) method. It predicts residue-residue contacts from multiple sequence alignments | <https://github.com/soedinglab/CCMpred> |
| pycoevol (Madeira and Krippahl, 2012) | A Python workflow to study Protein-protein Coevolution and interaction | <https://github.com/biomadeira/pycoevol> |
| CovET (Konecki et al., 2023) | Python tool that calculates coevolution scores by taking evolutionary divergence(Lichtarge et al., 1996) into account. | <https://github.com/LichtargeLab/Covariation-ET> |
| CIAlign (Tumescheit et al., 2022) | A highly customisable command line tool to clean, interpret and visualise multiple sequence alignments | <https://github.com/KatyBrown/CIAlign> |
| DCA-MOL(Jarmolinska et al., 2019) | A PyMol plugin to analyze coevolution data | <https://github.com/dzarmola/dca-mol> |
| pySCA(Rivoire et al., 2016) | Python implementation of statistical coupling analysis | <https://github.com/ranganathanlab/pySCA> |
| gaussian DCA(Baldassi et al., 2014) | Multivariate gaussian approach to calculate coevolution scores | <https://github.com/carlobaldassi/GaussDCA.jl> |
| nested coevolution (Colavin et al., 2022) | Adjusts coevolution scores based on phylogenetic information from inter- and intra-clade sequence comparisons. | <https://github.com/acolavin/nested-coevolution> |
| FreeContact(Kaján et al., 2014) | Fast implantation of mean field DCA and PSICOV | <https://www.cs.cit.tum.de/en/bio/home/> |
| **Web Servers** | | |
| GREMLIN | Web server interface for GREMLIN, allowing users to predict contacts and couplings from protein sequence alignments. | <https://gremlin.bakerlab.org/submit.php> |
| EVcouplings Web Server | Predicts coevolution scores and tertiary structure from multiple sequence alignments | <https://evcouplings.org/> |
| CAPS 2.0 (Fares and McNally, 2006) | Webserver to run coevolution analysis of proteins. | <http://bioinf.gen.tcd.ie/~faresm/software/software.html#caps> |
| MISTIC2 (Colell et al., 2018) | Allows users to run coevolution analysis using any of the following methods: mutual information, mfDCA, plmDCA, gaussianDCA, and PSICOV and analyse the results. | <https://mistic2.leloir.org.ar/#/introduction> |
| CoeViz (Baker and Porollo, 2016) | A web-based tool for coevolution analysis of proteins using one of the following statistical methods: Chi-square statistic, Pearson correlation, or Mutual Information. | <https://research.cchmc.org/CoevLab/coeviz2_help.html#About> |
| BIS2Analyzer(Oteri et al., 2017) | A tool for the online analysis of coevolving amino-acid pairs in protein sequences using blocks in sequences method (Dib and Carbone, 2012), specifically designed for vertebrate and viral proteins(Kaján et al., 2014; Oteri et al., 2022; De Oliveira et al., 2017) | <http://www.lcqb.upmc.fr/BIS2Analyzer/> |
| DCA server(Morcos et al., 2011) | Web server to run direct coupling analysis | <http://dca.rice.edu/> |
| ELIHKSIR(Sinner et al., 2021) | Predicts interactions sites using DCA between two proteins | <https://elihksir.org/> |
| iBIS2Analyzer (Oteri et al., 2022) | Server for phylogeny-based coevolution analysis | <http://ibis2analyzer.lcqb.upmc.fr/> |
| **Database Resources** | | |
| EVCouplings | EVCouplings provides access to precomputed evolutionary couplings and contact predictions for ~7,000 human proteins.  Lacks an API; allows querying for results only through UniProt or PFAM Accession ID’s. | <https://v1.evcouplings.org/precomputed/search> |
| GREMLIN | Contains contact predictions for 9846 bacterial proteins from four bacterial species listed below:   - [E. coli](https://gremlin2.bakerlab.org/preds.php?db=ECOLI): 3596 proteins - [B. subtilis](https://gremlin2.bakerlab.org/preds.php?db=BACSU): 3209 proteins - [H. salinarum](https://gremlin2.bakerlab.org/preds.php?db=HALSA): 1473 proteins - [S. solfataricus](https://gremlin2.bakerlab.org/preds.php?db=SULSO): 1568 proteins   Lacks an API; allows querying for results only through UniProt ID, gene or protein name. | <https://gremlin2.bakerlab.org/> |

**Supplementary Table 2:** List of features that can be queried from PyCoMdb.

| **Feature** | **Description** | **Query keyword** | | **Example** | **Additional information** |
| --- | --- | --- | --- | --- | --- |
|  |  | **Local/Remote API** | **Web API** |  |  |
| UniProt ID | UniProt ID of the protein | ID | uniprot_id | P0C9F6 |  |
| Sequence | Amino acid sequence | SEQUENCE | sequence | AKLMPALTYDGHA… | Partial match is not supported |
| Sequence length | Length of the aminoacid sequence | MIN_LENGTH | min_length | 100 |  |
|  |  | MAX_LENGTH | max_length | 350 |  |
| N_eff_ | Number of effective sequences in the alignment | - | - |  |  |
| **Structure properties** | | | | | |
| Helix (%) | % of helix content in the structure | MIN_HELIX | min_helix | 2 |  |
|  |  | MAX_HELIX | max_helix | 75 |  |
| Turn (%) | % of turn content in the structure | MIN_TURN | min_turn | 5 |  |
|  |  | MAX_TURN | max_turn | 10 |  |
| Strand (%) | % of strand content in the structure | MIN_STRAND | min_strand | 30 |  |
|  |  | MAX_STRAND | max_strand | 40 |  |
| PDB | PDB ID’s of known structures | HAS_PDB^1^ | has_pdb^1^ | True/False |  |
| Substrate | Whether the protein has a known substrate | HAS_SUBSTRATE^1^ | has_substrate^1^ | True/False |  |
| CATH ID | CATH classification of the protein | CATH | cath | 3.40.50.360 or 3.40.*.* or 3.* |  |
| Cofactor | Cofactors associated with the proteins | COFACTOR | cofactor | Zn(2+) | use pyc.get_cofactor_list() to get full list of co-factors |
| Cofactor ID | ID of the cofactors from CHEBI | COFACTOR_ID | cofactor_id | CHEBI:00001 | use pyc.get_cofactor_list() to get full list of co-factors |
| Domain | Domain associated with the protein | DOMAIN | domain | zinc-finger | use pyc.get_domain_list() for full list |
| Ligand | Ligand associated with the protein | LIGAND | ligand | glucose | pyc.get_ligand_list() for full list |
| **Modifications** | | | | | |
| PTM | Post-translational modification associated with the protein | PTM | ptm | phosphoprotein | use pyc.get_ptm_list() for full list |
|  | Whether the protein has a known post-translational modification | HAS_PTM^1^ | has_ptm^1^ | True/False |  |
| **Biological features** | | | | | |
| Organism ID | NCBI taxonomy ID of the genus/species | ORGANISM_ID | organism_id |  |  |
| Organism | Name of the genus/species | ORGANISM^*,2^ | organism^*,2^ | :homo: or homo | pyc.get_organism_list() for full list); Surround with : to get precise results, for example :homo: returns Homo sapiens & Homo sapiens neanderthalensis), while homo also returns **homo**eomma, t**homo**mys, and *hundreds* others |
| Enzyme Commission number | Enzyme Commission number of the protein | ENZYME | enzyme | 1.3.1.3 or 1.3.*.* or 1.* |  |
| Biological Process | Biological process associated with the protein | BIOLOGICAL_PROCESS | biological_process | antiviral defense | use pyc.get_biological_process_list() for full list |
| Cellular Component | Cellular component associated with the protein | CELLULAR_COMPONENT | cellular_component | nucleus | use pyc.get_cellular_component_list() for full list |
| Developmental Stage |  | DEVELOPMENTAL_STAGE |  |  |  |
| Molecular Function | Molecular function associated with the protein | MOLECULAR_FUNCTION | molecular_function | antioxidant activity | use pyc.get_molecular_function_list() for full list |
| Disease | The disease linked with the protein | DISEASE^*^ | disease^*^ | cancer | pyc.get_disease_list() to get full list of diseases |
|  |  | HAS_DISEASE^1^ | has_disease^1^ | True/False |  |
| Disease ID | The ID of the disease associated with the protein | DISEASE_ID | disease_id | DI-02205 | pyc.get_disease_list() to get full list of diseases |

^*^case-insensitive

**Supplementary Table 3:** Difference in functional features between the PyCoM library and WebAPI.

| Functionality | PyCoM Library | |  |
| --- | --- | --- | --- |
|  | Database is Local | Remote database on [https://pycom.brunel.ac.uk](https://pycom.brunel.ac.uk/) | Web API |
| Query the database | Y | Y | Y |
| Load matrices | Y | Y | Y |
| Display basic properties of each entry^1^ | Y | Y | Y |
| Provides access to   1. Diseases 2. CATH 3. EC ID 4. PDB id list 5. Organism taxonomy 6. Substrates list 7. List of Cofactors 8. PTM’s 9. List of Ligands 10. Molecular functions 11. biological processes 12. cellular components 13. protein domain 14. coding sequence diversity 15. developmental stage | Y | N | N |

^1^Uniprot_ID,N_eff_, sequence_length, sequence, Organism_ID, fraction of helix, turn, and strand, has_ptm, has_pdb, has_substrate, matrix


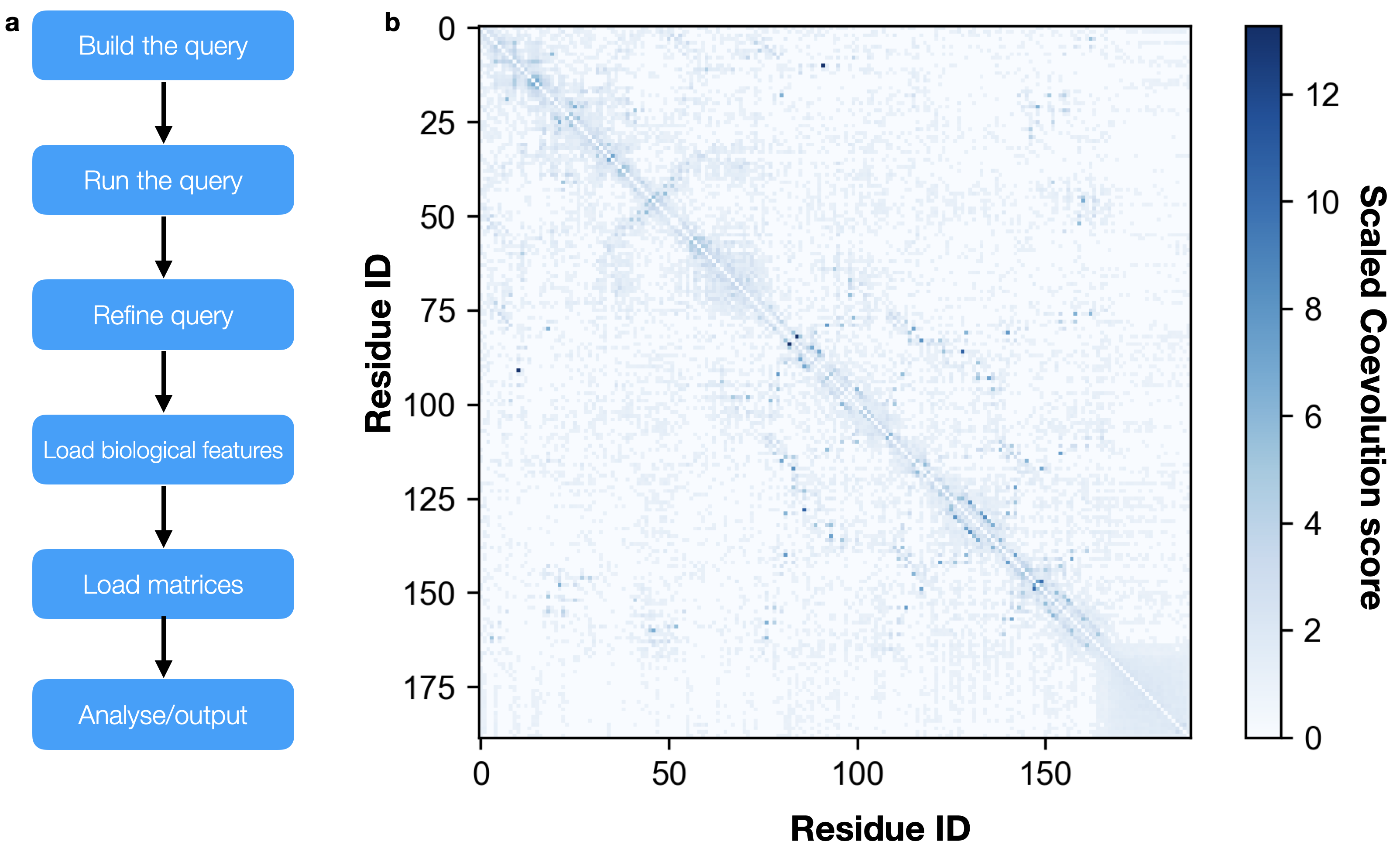


**Supplementary Figure 1:** a) Flowchart describing a typical workflow of how to use either the remote or local versions of PyCoM. b) Scaled coevolution matrix of GTPase NRas, UniProt ID P01111.

**References**

Baker,F.N. and Porollo,A. (2016) CoeViz: a web-based tool for coevolution analysis of protein residues. *BMC Bioinformatics*, **17**, 119.

Baldassi,C. *et al.* (2014) Fast and accurate multivariate Gaussian modeling of protein families: predicting residue contacts and protein-interaction partners. *PLoS One*, **9**, e92721.

Colavin,A. *et al.* (2022) Extracting phylogenetic dimensions of coevolution reveals hidden functional signals. *Sci Rep*, **12**.

Colell,E.A. *et al.* (2018) MISTIC2: Comprehensive server to study coevolution in protein families. *Nucleic Acids Res*, **46**, W323–W328.

Dib,L. and Carbone,A. (2012) Protein Fragments: Functional and Structural Roles of Their Coevolution Networks. *PLoS One*, **7**.

Fares,M.A. and McNally,D. (2006) CAPS: coevolution analysis using protein sequences. *Bioinformatics*, **22**, 2821–2.

Hopf,T.A. *et al.* (2017) Mutation effects predicted from sequence co-variation. *Nat Biotechnol*, **35**, 128–135.

Hopf,T.A. *et al.* (2019) The EVcouplings Python framework for coevolutionary sequence analysis. *Bioinformatics*, **35**, 1582–1584.

Jarmolinska,A.I. *et al.* (2019) DCA-MOL: A PyMOL Plugin To Analyze Direct Evolutionary Couplings. *J Chem Inf Model*, **59**, 625–629.

Jones,D.T. *et al.* (2015) MetaPSICOV: Combining coevolution methods for accurate prediction of contacts and long range hydrogen bonding in proteins. *Bioinformatics*, **31**, 999–1006.

Jones,D.T. *et al.* (2012) PSICOV: Precise structural contact prediction using sparse inverse covariance estimation on large multiple sequence alignments. *Bioinformatics*, **28**, 184–190.

Kaján,L. *et al.* (2014) FreeContact: fast and free software for protein contact prediction from residue co-evolution. *BMC Bioinformatics*, **15**, 85.

Kamisetty,H. *et al.* (2013) Assessing the utility of coevolution-based residue–residue contact predictions in a sequence- and structure-rich era. *Proceedings of the National Academy of Sciences*, **110**, 15674.

Konecki,D.M. *et al.* (2023) CovET: A covariation-evolutionary trace method that identifies protein structure–function modules. *Journal of Biological Chemistry*, **299**, 104896.

Lartillot,N. and Poujol,R. (2011) A phylogenetic model for investigating correlated evolution of substitution rates and continuous phenotypic characters. *Mol Biol Evol*, **28**, 729–744.

Lichtarge,O. *et al.* (1996) An Evolutionary Trace Method Defines Binding Surfaces Common to Protein Families.

Madeira,F. and Krippahl,L. (2012) Pycoevol: A Python workflow to study protein-protein coevolution. In, *BIOINFORMATICS 2012 - Proceedings of the International Conference on Bioinformatics Models, Methods and Algorithms*., pp. 143–149.

Martin,L.C. *et al.* (2005) Using information theory to search for co-evolving residues in proteins. *Bioinformatics*, **21**, 4116–4124.

Morcos,F. *et al.* (2011) Direct-coupling analysis of residue coevolution captures native contacts across many protein families. *Proceedings of the National Academy of Sciences*, **108**.

De Oliveira,S.H.P. *et al.* (2017) Comparing co-evolution methods and their application to template-free protein structure prediction. *Bioinformatics*, **33**, 373–381.

Oteri,F. *et al.* (2017) BIS2Analyzer: a server for co-evolution analysis of conserved protein families. *Nucleic Acids Res*, **45**, W307–W314.

Oteri,F. *et al.* (2022) iBIS2Analyzer: a web server for a phylogeny-driven coevolution analysis of protein families. *Nucleic Acids Res*, **50**, W412–W419.

Rivoire,O. *et al.* (2016) Evolution-Based Functional Decomposition of Proteins. *PLoS Comput Biol*, **12**, e1004817.

Seemayer,S. *et al.* (2014) CCMpred - Fast and precise prediction of protein residue-residue contacts from correlated mutations. *Bioinformatics*, **30**, 3128–3130.

Sinner,C. *et al.* (2021) ELIHKSIR Web Server: Evolutionary Links Inferred for Histidine Kinase Sensors Interacting with Response Regulators. *Entropy (Basel)*, **23**.

Steinegger,M. *et al.* (2019) HH-suite3 for fast remote homology detection and deep protein annotation. *BMC Bioinformatics*, **20**.

Tumescheit,C. *et al.* (2022) CIAlign: A highly customisable command line tool to clean, interpret and visualise multiple sequence alignments. *PeerJ*.

Zerihun,M.B. *et al.* (2020) Pydca v1.0: A comprehensive software for direct coupling analysis of RNA and protein sequences. *Bioinformatics*, **36**, 2264–2265.
