# Supplementary material for: PyCoM: a python library for large-scale analysis of residue–residue coevolution data
Source: Bioinformatics. 2024 Mar 26;40(4):btae166. doi: 10.1093/bioinformatics/btae166 (PMC11009027; doi:10.1093/bioinformatics/btae166)
Supplement: btae166_Supplementary_Data [file btae166_supplementary_data.zip › 04_Paper_Images.pdf]

Generate images from the paper

In [ ]:
```
# importing all usefull classes from PyCoM
from pycom import PyCom, ProteinParams,CoMAnalysis
import pandas as pd
import numpy as np
# matplotlib
import matplotlib
import matplotlib.pyplot as plt
matplotlib.rcParams['pdf.fonttype'] = 42
matplotlib.rcParams['font.family'] = "sans-serif"
matplotlib.rcParams['font.sans-serif'] = "Arial"
```

In [ ]:
```
#set the path to the database
database_folder_path="/Volumes/mason/Work/Sarath/Research/pycom/"
file_matrix_db = database_folder_path+"pycom.mat"
file_protein_db= database_folder_path+"pycom.db"
my_color="#6495ED"
```

In [ ]:
```
obj_pycom = PyCom(db_path=file_protein_db, mat_path=file_matrix_db)
```

In [ ]:
```
obj_pycom = PyCom(remote=True)
```

Construct your query (its empty as I want all information)

In [ ]:
```
# Here we are asking for all the proteins that match the enzyme class 3 a
query_parameters={}
# executing the query returns a pandas dataframe with information about a
```

Finding out dimensions of the dataframe:

In [ ]:
```
entries_data_frame=obj_pycom.find(query_parameters)
```

```
/Users/sdantu/Work/pyc_wspace/pycom/pycom/pycom/interface/_find_helper.py:
19: UserWarning: No constraints were passed to find(). This will return al
l proteins in the database.
  warn('No constraints were passed to find(). This will return all protein
s in the database.')
```

In [ ]:
```
entries_data_frame.describe()
```

Out[ ]:

	neff	sequence_length	helix_frac	turn_frac	stran[illegible]
**count**	457622.000000	457622.000000	457622.000000	457622.000000	457622.0
**mean**	8.397407	251.278734	0.013926	0.001262	0.0
**std**	2.498266	124.627642	0.076069	0.008348	0.[illegible]
**min**	1.000000	5.000000	0.000000	0.000000	0.0
**25%**	6.928000	147.000000	0.000000	0.000000	0.0
**50%**	8.621000	243.000000	0.000000	0.000000	0.0
**75%**	10.176000	351.000000	0.000000	0.000000	0.0
**max**	17.205000	500.000000	0.956522	0.542857	0.[illegible]

Save the query to a csv file

In [ ]:
```
entries_data_frame.to_csv("Full_DB_Query.csv",index=False)
```

Read query data from csv file

In [ ]:
```
#entries_data_frame=pd.read_csv("Full_DB.csv")
```

Find unique entries in a column:

In [ ]:
```
entries_data_frame['has_ptm'].unique()
```

In [ ]:
```
entries_data_frame['has_pdb'].value_counts()
```

In [ ]:
```
entries_data_frame["neff"].min()
```

Supported query keywords:

- `uniprot_id` : The UniProt ID of the protein.
- `sequence` : The amino acid sequence of protein to search for. (full match)
- `min_length` / `max_length` : Min/Max number of residues in the protein.
- `min_helix` / `max_helix` : Min/Max percentage of helical structure in the protein.
- `min_turn` / `max_turn` : Min/Max percentage of turn structure in the protein.
- `min_strand` / `max_strand` : Min/Max percentage of beta strand structure in the protein.
- `organism` : Taxonomic name of the genus / species of the protein. (case-insensitive)

        - Species name or any parent taxonomic level can be used. (`pyc.get_organism_list()` for full list)
        - Surround with `:` to get precise results
            - `:homo:` returns `Homo sapiens` & `Homo sapiens neanderthalensis`)
            - `homo` also returns **homo**eomma, t**homo**mys, and *hundreds* others
- `organism_id`: Precise NCBI Taxonomy ID of the species of the protein. (prefer to use `organism` instead)
- `cath`: CATH classification of the protein (`3.40.50.360` or `3.40.*.*` or `3.*`).
- `enzyme`: Enzyme Commission number of the protein. (`1.3.1.3` or `1.3.*.*` or `1.*`).
- `has_substrate`: Whether the protein has a known substrate. (`True`/`False`)
- `has_ptm`: Whether the protein has a known post-translational modification. (`True`/`False`)
- `has_pbd`: Whether the protein has a known PDB structure. (`True`/`False`)
- `disease`: The disease associated with the protein. (name of disease, case-insensitive, e.g `cancer`)
    - Use `pyc.get_disease_list()` for full list.
    - `cancer` searches for `Ovarian cancer`, `Lung cancer`, ...
- `disease_id`: The ID of the disease associated with the protein. (`DI-02205`, get_disease_list()
- `has_disease`: Whether the protein is associated with a disease. (`True`/`False`)
- `cofactor`: The cofactor associated with the protein. (name of cofactor, case-insensitive, e.g `Zn(2+)`])
- `cofactor_id`: The ID of the cofactor associated with the protein. (`CHEBI:00001`, get_cofactor_list())
- `biological_process`: Biological process associated with the protein. (e.g `antiviral defense`, use `pyc.get_biological_process_list()` for full list)
- `cellular_component`: Cellular component associated with the protein. (e.g `nucleus`, use `pyc.get_cellular_component_list()` for full list
- `domain`: Domain associated with the protein. (e.g `zinc-finger`, use `pyc.get_domain_list()` for full list)
- `ligand`: Ligand associated with the protein. (e.g `zinc`, use `pyc.get_ligand_list()` for full list
- `molecular_function`: Molecular function associated with the protein. (e.g `antioxidant activity`, use `pyc.get_molecular_function_list()` for full list

- `ptm` : Post-translational modification associated with the protein. (e.g `phosphoprotein` , use `pyc.get_ptm_list()` for full list

Here is an example of making a large query, then paginating the results:

In [ ]:
```
# plotting parameters
ticks_font=12
labels_font=14
```

Plot N_eff

In [ ]:
```
xlabel='$\mathrm{N}_{eff}$'
ylabel="Count"
plt.figure(figsize=(4,3))
neff_hist=plt.hist(entries_data_frame["neff"],bins=50,color=my_color,cumu
plt.xlabel(xlabel,fontsize=labels_font)
plt.ylabel(ylabel,fontsize=labels_font)
plt.xticks(np.arange(0,18,2),fontsize=ticks_font)
plt.yticks(fontsize=ticks_font)
plt.grid(linestyle="--",lw=1)
plt.tight_layout()
plt.savefig("Neff.png",dpi=300,transparent=True)
```

Plot Sequence length distribution

In [ ]:
```
xlabel='Sequence length'
ylabel="Count"
plt.figure(figsize=(4,3))
seq_hist=plt.hist(entries_data_frame["sequence_length"],bins=50,color=my_
plt.xlabel(xlabel,fontsize=labels_font)
plt.ylabel(ylabel,fontsize=labels_font)
plt.xticks(np.arange(0,550,100),fontsize=ticks_font)
plt.yticks(fontsize=ticks_font)
plt.grid(linestyle="--",lw=1)
plt.tight_layout()
plt.savefig("seq_len.png",dpi=300,transparent=True)
```

In [ ]:
```
#This is not informative

xlabel='Secondary structure (%)'
ylabel="Count"
helix_hist=plt.hist(entries_has_pdb["helix_frac"]*100,bins=50,color="#649
strand_hist=plt.hist(entries_has_pdb["strand_frac"]*100,bins=50,color="#F
turn_hist=plt.hist(entries_has_pdb["helix_frac"]*100,bins=50,color="#DE31

plt.xlabel(xlabel,fontsize=labels_font)
plt.ylabel(ylabel,fontsize=labels_font)
plt.xticks(np.arange(0,110,10),fontsize=ticks_font)
```

```
plt.yticks(fontsize=ticks_font)
plt.savefig("sstruc.png",dpi=300,transparent=True)
```

Get columns in the dataframe

In [ ]:
```
entries_data_frame.head()
```

Add biological features to the dataframe for each protein

Initialise the object loader class and then call each add function

1. Add Enzyme Classification
2. Add CATH Class
3. Add Co-factors
4. Add PTM
5. Add Diseases

In [ ]:
```
obj_data_loader=obj_pycom.get_data_loader()
entries_data_frame=obj_data_loader.add_enzyme_commission(entries_data_fra
entries_data_frame=obj_data_loader.add_cath_class(entries_data_frame,forc
```

In [ ]:
```
entries_data_frame=obj_data_loader.add_pdbs(entries_data_frame,force_sing
```

In [ ]:
```
entries_data_frame=obj_data_loader.add_cofactors(entries_data_frame,force
```

In [ ]:
```
entries_data_frame=obj_data_loader.add_ptm(entries_data_frame,force_singl
```

In [ ]:
```
entries_data_frame=obj_data_loader.add_diseases(entries_data_frame,force_
```

Save the progress to a csv file

In [ ]:
```
entries_data_frame.to_csv("Full_DB_With_Details.csv",index=False)
```

In [ ]:
```
entries_data_frame['sequence'].unique().sum()
```

Number of entries with PDB files available

In [ ]:
```
entries_data_frame["pdb_id"].notna().sum()
```

In [ ]:
```
ec_full.describe(include="all")
```

In [ ]:
```
entries_data_frame["cofactor"].notna().sum()
```

In [ ]:
```
entries_data_frame["cath_class"].notna().sum()
```

In [ ]:
```
entries_with_ec_data=entries_data_frame[entries_data_frame["enzyme_commis
entries_with_cath_data=entries_data_frame[entries_data_frame["cath_class"
```

In [ ]:
```
entries_with_cath_data["cath_class"].isna().value_counts()
```

In [ ]:
```
from collections import OrderedDict

def group_data_by_class(data,data_type=1):
    global dict_group_data
    dict_group_data={}

    data_class="enzyme_commission"
    if(data_type==1):
        data_class="enzyme_commission"
    if(data_type==2):
        data_class="cath_class"

    for i_data in data[data_class]:
        n=len(i_data)
        if(n==1):
            classid=i_data[0].split('.')[0]
            update_dict_group(classid)
        if(n>1):
            for j in i_data:
                update_dict_group(j[0].split('.')[0])
    dict_group_data=dict(sorted(dict_group_data.items()))
    return dict_group_data
def update_dict_group(classid):
    global dict_group_data

    if(classid in dict_group_data.keys()):
        dict_group_data[classid]=dict_group_data[classid]+1
    else:
        dict_group_data[classid]=1
```

In [ ]:
```
ec_numbers=group_data_by_class(entries_with_ec_data,data_type=1)
ec_numbers
```

In [ ]:
```
cath_numbers=group_data_by_class(entries_with_cath_data,data_type=2)
cath_numbers
```

In [ ]:
```
entries_with_cath_data["cath_class"].isna().sum()
```

In [ ]:
```
entries_with_cath_data
```

In [ ]:
```
ec_pie=plt.pie(ec_numbers.values(),
               labels=ec_numbers.keys(),
               autopct='%1.1f%%',
               textprops=dict(color="w",fontsize=10),
               startangle=90)
plt.savefig("ecdata.png",dpi=300,transparent=True)
```

In [ ]:
```
cath_pie=plt.pie(cath_numbers.values(),
                 labels=cath_numbers.keys(),
                 autopct='%1.1f%%',
                 textprops=dict(color="black",fontsize=10),
                 startangle=90)
plt.tight_layout()
plt.savefig("cathdata.png",dpi=300,transparent=True)
```

In [ ]:
```
plt.figure(figsize=(4,3))
plt.bar(cath_numbers.keys(),
                   height=cath_numbers.values(),
                   color=my_color
                  )
plt.xlabel("CATH Class",fontsize=labels_font)
plt.ylabel("Count",fontsize=labels_font)
plt.xticks(fontsize=ticks_font)
plt.yticks(fontsize=ticks_font)
plt.grid(axis='y',ls="--",lw=1)
plt.tight_layout()
plt.savefig("cathdata.png",dpi=300,transparent=True)
```

In [ ]:
```
plt.figure(figsize=(4,3))
plt.bar(ec_numbers.keys(),
        height=ec_numbers.values(),
        color=my_color,
       )
plt.xlabel("Enzyme Commission Class",fontsize=labels_font)
plt.ylabel("Count",fontsize=labels_font)
plt.xticks(fontsize=ticks_font)
plt.yticks(fontsize=ticks_font)
plt.grid(axis='y',ls="--",lw=1)
plt.tight_layout()
plt.savefig("ecdata.png",dpi=300,transparent=True)
```

In [ ]:
```
dis=entries_data_frame[entries_data_frame['enzyme_commission'].notna()]
```

In [ ]:
```
dis["enzyme_commission"].unique()
```

In [ ]:
```
Protein
```
