# Supplementary material for: PyCoM: a python library for large-scale analysis of residue–residue coevolution data
Source: Bioinformatics. 2024 Mar 26;40(4):btae166. doi: 10.1093/bioinformatics/btae166 (PMC11009027; doi:10.1093/bioinformatics/btae166)
Supplement: btae166_Supplementary_Data [file btae166_supplementary_data.zip › 01_Workflow.pdf]

Workflow of PyCoM

In this tutorial, with a small example, you will learn how to create a workflow with the local version of PyCoM, using the steps listed below:

1. Setup
2. Initalise pycom objects
3. Create a query dictionary
4. Save and retrieve progress
5. Analyse search results
6. Add biological features
7. Some statistics
8. Coevolution matrix analysis
9. Help on UniProt Controlled Vocabulary

Setup

The assumption is that you have completed the installation and downloaded the database. For help on this please look at the quick guide here.

Initalise pycom objects

First, lets import all the libraries and classes we need from pycom, pandas, matplotlib, and numpy

In [ ]:
```
# importing all usefull classes from PyCoM
from pycom import PyCom, ProteinParams,CoMAnalysis
import pandas as pd
import numpy as np
# matplotlib; useful for plotting later
import matplotlib
import matplotlib.pyplot as plt
#setting matplotlib parameters
matplotlib.rcParams['pdf.fonttype'] = 42
matplotlib.rcParams['font.family'] = "sans-serif"
matplotlib.rcParams['font.sans-serif'] = "Arial"
```

In [ ]:
```
#set the path to the database
database_folder_path="/Volumes/mason/Work/Sarath/Research/pycom/"
#matrix file name and path
file_matrix_db = database_folder_path+"pycom.mat"
#protein database file name and path
file_protein_db= database_folder_path+"pycom.db"
```

In [ ]:
```
obj_pycom = PyCom(db_path=file_protein_db, mat_path=file_matrix_db)
```

Create a query dictionary

To query the database, we need to create a dictionary object `query_parameters` using the keywords for our choice of properties. For the full list of keywords please check

Empty `query_parameters` will return information on all the ~457,000 proteins in the database

To query the database, we need to create a dictionary object `query_parameters` using the keywords for our choice of properties. For the full list of keywords please check

Empty `query_parameters` will return information on all the ~457,000 proteins in the database

In [ ]:
```
#creating empty query dictionary
query_parameters={}
```

In [ ]:
```
# Here we are asking for all the proteins that match the enzyme class 3 a
query_parameters={ProteinParams.DISEASE:"cancer",
                  ProteinParams.ENZYME: '3.*',
                  ProteinParams.MIN_LENGTH: 100,
                  ProteinParams.MAX_LENGTH: 200,
                 }
```

Executing the query with the parameters defined in the above cell using the pycom object `obj_pycom` `find()` function will return a pandas dataframe with the search results containing information about all the proteins which match our query.

In [ ]:
```
entries_data_frame=obj_pycom.find(query_parameters)
```

Save and retrieve progress

We can save and retreive our progress by saving our dataframe with information on our favourite proteins by saving it to a csv file.

Save the query to a csv file

To avoid rerunning the query we can cave the progress to a csv file.

In [ ]:
```
entries_data_frame.to_csv("output/DB_Query_Results.csv",index=False)
```

Read query data from csv file

Retrieving our progress from the csv file.

In [ ]:
```
#entries_data_frame=pd.read_csv("output/DB_Query_Results.csv")
```

Analyse search results

The search returns a pandas data frame with proteins matching the query critiera with following information for each protein:

- uniprot_id: Uniprot ID
- neff: Depth of the sequence alignment N_{eff}
- sequence_length: Sequence length
- sequence: protein sequence
- organism_id: Organism ID
- helic_frac, turn_frac, strand_frac: helix, turn, and strand structure fraction
- has_ptm: Has a PTM Yes/No
- has_pdb: Has a PDB structure Yes/No
- has_substrate: Has a substrate for biological activity Yes/No
- matrix: coevolution matrix column is empty because at this stage we would still want you to check the search results and if required filter them based on any of the biological properties before loading the matrices.

First, look what columns we have and their names.

In [ ]:
```
entries_data_frame.head()
```

Out[ ]:

	uniprot_id	neff	sequence_length	s
0	P01111	12.817	189	MTEYKLVVVGAGGVGKSALTIQLIQNHFVDEYDPTIEDSYI
1	P01112	12.841	189	MTEYKLVVVGAGGVGKSALTIQLIQNHFVDEYDPTIEDSYI
2	P01116	12.626	189	MTEYKLVVVGAGGVGKSALTIQLIQNHFVDEYDPTIEDSYI

`describe()` function from pandas can be used to get a summary of all the features:

In [ ]:
```
entries_data_frame.describe(include="all")
```

Out[ ]:

	uniprot_id	neff	sequence_length	
**count**	3	3.000000	3.0	
**unique**	3	NaN	NaN	
**top**	P01111	NaN	NaN	MTEYKLVVVGAGGVGKSALTIQLIQNHFVDEYD
**freq**	1	NaN	NaN	
**mean**	NaN	12.761333	189.0	
**std**	NaN	0.117815	0.0	
**min**	NaN	12.626000	189.0	
**25%**	NaN	12.721500	189.0	
**50%**	NaN	12.817000	189.0	
**75%**	NaN	12.829000	189.0	
**max**	NaN	12.841000	189.0	

Get counts of categorical data in the column, for example:

- number of proteins with a known PDB structure
- number of proteins with a known PTM

In [ ]:

```
entries_data_frame['has_pdb'].value_counts()
```

Out[ ]:

```
1    3
Name: has_pdb, dtype: int64
```

Find number of unique elements in the column, for example number of unique organisms:

In [ ]:

```
entries_data_frame['organism_id'].unique()
```

Out[ ]:

```
array(['9606'], dtype=object)
```

All the sequences are from the same organism, `9606` i.e. from `Homo sapiens`. Full list is available from UniProt

Some statistics on the numerical column, for example `neff`:

In [ ]:

```
entries_data_frame["neff"].describe()
```

Out[ ]:
```
count     3.000000
mean     12.761333
std       0.117815
min      12.626000
25%      12.721500
50%      12.817000
75%      12.829000
max      12.841000
Name: neff, dtype: float64
```

We can also use other functions to get some of the information

In [ ]:
```
entries_data_frame["neff"].min()
```

Out[ ]:
```
12.626
```

In [ ]:
```
entries_data_frame["neff"].mean()
```

Out[ ]:
```
12.761333333333333
```

Add biological features

Initialise the object loader class and then call each add function

1. Add Enzyme Classification
2. Add CATH Class
3. Add Co-factors
4. Add PTM
5. Add Diseases

For a protein entry if the requested data (EC/CATH/Cofactors...) does not exist, corresponding entry in that column will be `nan`. We can filter such rows as shown further below.

Please note the dataloader functions will not work with remote version of PyCoM

In [ ]:
```
#initialise the object for data loader class
obj_data_loader=obj_pycom.get_data_loader()
```

In [ ]:
```
#add enzyme commission data to the dataframe
entries_data_frame=obj_data_loader.add_enzyme_commission(entries_data_fra
#add CATH data to the dataframe
entries_data_frame=obj_data_loader.add_cath_class(entries_data_frame,forc
#add CATH data to the dataframe
entries_data_frame=obj_data_loader.add_pdbs(entries_data_frame,force_sing
#get list of all cofactors for each protein
entries_data_frame=obj_data_loader.add_cofactors(entries_data_frame,force
#get list of all PTM's for each protein
entries_data_frame=obj_data_loader.add_ptm(entries_data_frame,force_singl
#get list of all diseases for each protein
entries_data_frame=obj_data_loader.add_diseases(entries_data_frame,force_
```

In [ ]:
```
#get substrates for the proteins
entries_data_frame=obj_data_loader.add_ligand(entries_data_frame,force_si
```

Save the progress to a csv file

As we have added a lot of information to our dataframe, let's save our progress so that we can restart from this point, in future, if required.

In [ ]:
```
entries_data_frame.to_csv("output/DB_Query_Results_With_Details.csv",inde
```

Some Statistics

Let's look at some statistics for all the columns in the dataframe. Below are some examples of how you can do some fun things with the dataframe.

In [ ]:
```
#include=all will also include columns with 'nan' entries
entries_data_frame.describe(include="all")
```

Out[ ]:

	uniprot_id	neff	sequence_length	
count	3	3.000000	3.0	
unique	3	NaN	NaN	
top	P01111	NaN	NaN	MTEYKLVVVGAGGVGKSALTIQLIQNHFVDEYD
freq	1	NaN	NaN	
mean	NaN	12.761333	189.0	
std	NaN	0.117815	0.0	
min	NaN	12.626000	189.0	
25%	NaN	12.721500	189.0	
50%	NaN	12.817000	189.0	
75%	NaN	12.829000	189.0	
max	NaN	12.841000	189.0	

Find unique ligands and count them:

In [ ]:
```
entries_data_frame['ligand'].value_counts()
```

Out[ ]:
```
[GTP-binding, Nucleotide-binding]    3
Name: ligand, dtype: int64
```

In [ ]:
```
# Filter the search results where we have a ligand interacting with the p
df_results_with_ligand=entries_data_frame[entries_data_frame['ligand'].no
```

Count columns without 'nan' entries

In [ ]:
```
#Number of proteins with pdb data
df_results_with_ligand["pdb_id"].notna().sum()
```

Out[ ]:
```
3
```

Coevolution matrix analysis

Load the matrix

Lets get the coevolution matrix for the filtered dataframe `df_results_with_ligand` using the `load_matrices()` from `obj_pycom`

In [ ]:
```
df_results_with_ligand=obj_pycom.load_matrices(df_results_with_ligand)
```

In [ ]:
```
df_results_with_ligand
```

Out[ ]:

	uniprot_id	neff	sequence_length	s
0	P01111	12.817	189	MTEYKLVVVGAGGVGKSALTIQLIQNHFVDEYDPTIEDSYI
1	P01112	12.841	189	MTEYKLVVVGAGGVGKSALTIQLIQNHFVDEYDPTIEDSYI
2	P01116	12.626	189	MTEYKLVVVGAGGVGKSALTIQLIQNHFVDEYDPTIEDSYI

Normalise/Scale the matrix

Scaled matrix (S_i): Coevolution Matrices (C_i) have to be scaled by average $\langle C_i \rangle$, all values < $\langle C_i \rangle$ are set to 0.

Normalised matrix (N_i): For comparing scaled coevolution scores across multiple proteins, we can normalise the values of all matrices $S_i \ldots S_n$, by dividing them by the $\max(S_i \ldots S_n)$.

These operations can be performed by using *object* from `CoMAnalysis` class.

In [ ]:
```
#initialise CoMAnalysis class object
obj_com_analysis=CoMAnalysis()
```

In [ ]:
```
df_results_with_ligand_matrix=obj_com_analysis.scale_and_normalise_coevol
```

In [ ]:
```
df_results_with_ligand_matrix.head()
```

Out[ ]:

	uniprot_id	neff	sequence_length	s
0	P01111	12.817	189	MTEYKLVVVGAGGVGKSALTIQLIQNHFVDEYDPTIEDSYI
1	P01112	12.841	189	MTEYKLVVVGAGGVGKSALTIQLIQNHFVDEYDPTIEDSYI
2	P01116	12.626	189	MTEYKLVVVGAGGVGKSALTIQLIQNHFVDEYDPTIEDSYI

3 rows × 22 columns

`matrix_S` column contains the S_i matrix and the `matrix_N` column contains N_i

Plot the matrix and save it

Lets plot the matrix for the first `(index is 0)` protein in the dataframe.

In [ ]:
```
plt.imshow(df_results_with_ligand_matrix.loc[0,'matrix_S'],cmap='Blues')
plt.colorbar()
file_name="output/%s_Scaled_Matrix.png"%(df_results_with_ligand_matrix.lo
plt.savefig(file_name,dpi=300)
```

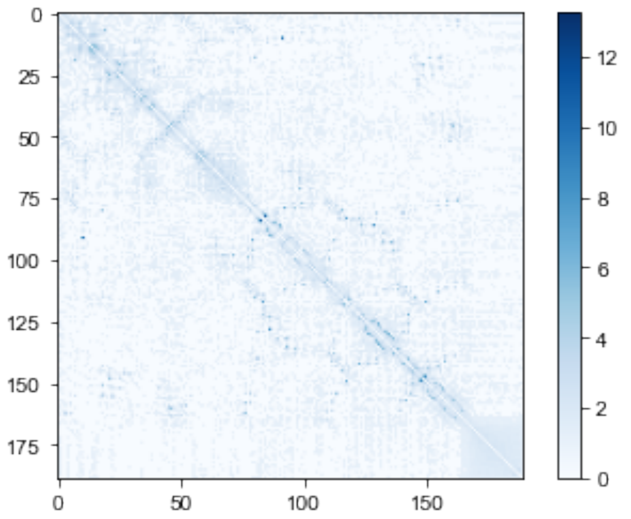

In [ ]:
```
plt.imshow(df_results_with_ligand_matrix.loc[0,'matrix_N'],cmap='Blues')
plt.colorbar()
file_name="output/%s_Normalised_Matrix.png"%(df_results_with_ligand_matri
plt.savefig(file_name,dpi=300)
```

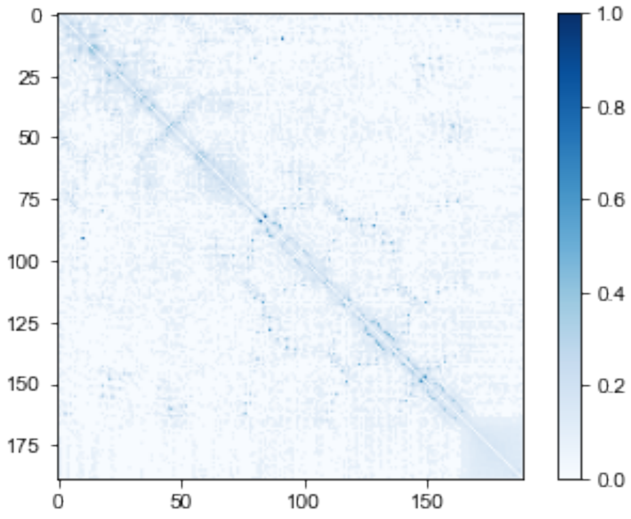

Save the list of top coevolution pairs

Residues with scores close to 1 have the strongest evolutionary relationship, and close to 0 may not be covarying during evolution.

The top scoring residues are sorted in descending order and saved to an ASCII file for further interpretation.

In [ ]:
```
obj_com_analysis.save_top_scoring_residue_pairs(df_results_with_ligand_ma
```

Help on UniProt Controlled Vocabulary

For each biological feature category, UniProtKB/Swiss-Prot has a curated list of keywords. To search using those keywords or ID's and help you find them we have some helper functions that will help you find them:

Function `get_cofactor_list()` from `pycom` will get you list of cofactors. You can either use the `cofactorId` or the `cofactorName`.

In [ ]:
```
# list of cofactors
cofactors = obj_pycom.get_cofactor_list()
cofactors
```

Out[ ]:

	cofactorId	cofactorName
**0**	CHEBI:597326	pyridoxal 5'-phosphate
**1**	CHEBI:18420	Mg(2+)
**2**	CHEBI:60240	a divalent metal cation
**3**	CHEBI:30413	heme
**4**	CHEBI:29105	Zn(2+)
**...**	...	...
**109**	CHEBI:61721	chlorophyll b
**110**	CHEBI:73095	divinyl chlorophyll a
**111**	CHEBI:73096	divinyl chlorophyll b
**112**	CHEBI:57453	(6S)-5,6,7,8-tetrahydrofolate
**113**	CHEBI:30402	tungstopterin

114 rows × 2 columns

Function `get_disease_list()` from `pycom` will get you list of diseases. You can either use the `diseaseId` or the `diseaseName`.

In [ ]:
```
# list of diseases
diseases = obj_pycom.get_disease_list()
diseases
```

Out[ ]:

	diseaseId	diseaseName
**0**	DI-04420	Intellectual developmental disorder, autosomal...
**1**	DI-00085	Alzheimer disease 1
**2**	DI-00097	Cerebral amyloid angiopathy, APP-related
**3**	DI-00262	Chanarin-Dorfman syndrome
**4**	DI-01042	Spastic paraplegia 42, autosomal dominant
**...**	...	...
**6039**	DI-05800	Wieacker-Wolff syndrome, female-restricted
**6040**	DI-01041	Spastic paraplegia 33, autosomal dominant
**6041**	DI-05703	Neurodevelopmental disorder with dysmorphic fa...
**6042**	DI-06050	Intellectual developmental disorder, autosomal...
**6043**	DI-04662	Paget disease of bone 6

6044 rows × 2 columns

Function `get_organism_list()` from `pycom` will get you list of diseases. You can either use the `organismId` or the `nameScientific` or `nameCommon` or any categories in the `taxonomy`.

In [ ]:

```
# list of organisms
organisms = obj_pycom.get_organism_list()
organisms
```

Out[ ]:

	organismId	nameScientific	nameCommon	
0	561445	African swine fever virus (isolate Pig/Kenya/K...	ASFV	:Viruses:Varidnaviria:Bamford
1	10500	African swine fever virus (isolate Tick/Malawi...	ASFV	:Viruses:Varidnaviria:Bamford
2	561443	African swine fever virus (isolate Tick/South ...	ASFV	:Viruses:Varidnaviria:Bamford
3	561444	African swine fever virus (isolate Warthog/Nam...	ASFV	:Viruses:Varidnaviria:Bamford
4	10498	African swine fever virus (strain Badajoz 1971...	Ba71V	:Viruses:Varidnaviria:Bamford
...	...	...	...	
14316	31581	Rotavirus A (isolate RVA/Pig/Australia/TFR-41/...	RV-A	:Viruses:Riboviria:Orthornav
14317	31579	Rotavirus A (isolate RVA/Pig/Australia/BEN144/...	RV-A	:Viruses:Riboviria:Orthornav
14318	10918	Rotavirus A (strain RVA/Pig/Russia/K/1987)	RV-A	:Viruses:Riboviria:Orthornav
14319	31580	Rotavirus A (isolate RVA/Pig/Australia/BMI-1/1...	RV-A	:Viruses:Riboviria:Orthornav
14320	47664	Populus tremula x Populus tremuloides	Hybrid aspen	:Eukaryota:Viridiplantae:Strept

14321 rows × 4 columns

In [ ]:

```
#full list of helper functions to get searchable terms on other biologica
obj_pycom.get_biological_process_list()
obj_pycom.get_cellular_component_list()
obj_pycom.get_developmental_stage_list()
obj_pycom.get_domain_list()
obj_pycom.get_ligand_list()
obj_pycom.get_ptm_list()
obj_pycom.get_molecular_function_list()
```

Out[ ]:

	name
**0**	Acetylcholine receptor inhibiting toxin
**1**	Actin-binding
**2**	Activator
**3**	Acyltransferase
**4**	Allosteric enzyme
**...**	...
**191**	Viral short tail ejection system
**192**	Viral exotoxin
**193**	Chloride channel impairing toxin
**194**	Proton-gated sodium channel impairing toxin
**195**	Translocase

196 rows × 1 columns
